# Surgical Management of Poorly Differentiated Thyroid Carcinoma Extending Into the Superior Vena Cava: A Case Report

**DOI:** 10.7759/cureus.97394

**Published:** 2025-11-20

**Authors:** Kotaro Terayama, Wataru Kida, Akiko Ito, Motoyuki Hisagi, Muneo Nakaya

**Affiliations:** 1 Department of Otolaryngology-Head and Neck Surgery, Tokyo Metropolitan Tama Medical Center, Tokyo, JPN; 2 Department of Cardiovascular Surgery, Tokyo Metropolitan Tama Medical Center, Tokyo, JPN

**Keywords:** lenvatinib, poorly differentiated thyroid carcinoma, superior vena cava (svc) obstruction, surgical oncology management, thyroid cancer surgery

## Abstract

Poorly differentiated thyroid carcinoma (PDTC) is a rare histological type with a poorer prognosis than well-differentiated thyroid carcinoma. Herein, we report a case of a tumor extending from the left thyroid lobe into the superior vena cava (SVC), which was successfully resected in a joint operation with cardiovascular surgeons.

A 75-year-old man was referred to our department after a left thyroid lobe tumor was incidentally found on a CT scan. Contrast-enhanced CT revealed that the entire left thyroid lobe was replaced by a tumor, which extended through the left inferior thyroid vein and brachiocephalic vein into the SVC. Bilateral cervical lymph node metastases and multiple pulmonary metastases were also observed. A needle biopsy led to a suspected diagnosis of PDTC.

Total thyroidectomy, bilateral neck dissection, and resection of the tumor in the SVC via thoracotomy were performed. The patient had an uneventful recovery and was discharged on postoperative day 9. The histopathological diagnosis of the surgical specimen confirmed PDTC. Postoperatively, radioactive iodine (RAI) therapy was considered, but it was not administered due to the absence of iodine uptake. Instead, treatment with lenvatinib was initiated. With lenvatinib treatment, the multiple pulmonary metastases have shown regression, and no recurrence has been observed locally, in the cervical lymph nodes, or in the SVC.

PDTC is rare, and only one other case report of PDTC extending into the SVC has been published. This case suggests that even for a tumor thrombus from poorly differentiated carcinoma, surgical resection can be a viable option if imaging studies show no evidence of invasion into the vessel wall.

## Introduction

Poorly differentiated thyroid carcinoma (PDTC) is a rare histological type with a poorer prognosis compared to a well-differentiated thyroid carcinoma. In 2004, PDTC was recognized as a distinct pathologic entity in the World Health Organization (WHO) classification of endocrine tumors [1,2]. Subsequently, in 2007, the diagnosis of PDTC became more stringent under the Turin criteria [3]. From a clinical standpoint, tumor thrombus formation in the vena cava is rare in thyroid cancer, and only a few reports describe thyroid cancer patients in whom tumor thrombus extended beyond the internal jugular vein into the brachiocephalic vein and superior vena cava (SVC) [4-6]. This report describes a case of PDTC originating in the left thyroid lobe and extending into the SVC, which was successfully resected via radical resection through a combined surgical approach with the cardiovascular surgery team.

## Case presentation

A 75-year-old man, who had been aware of a neck mass for two years, was admitted to the Department of Gastroenterology at our hospital for a duodenal ulcer. A thyroid tumor was identified during a CT scan performed at that time, and he was subsequently referred to our department.

**Figure 1:**
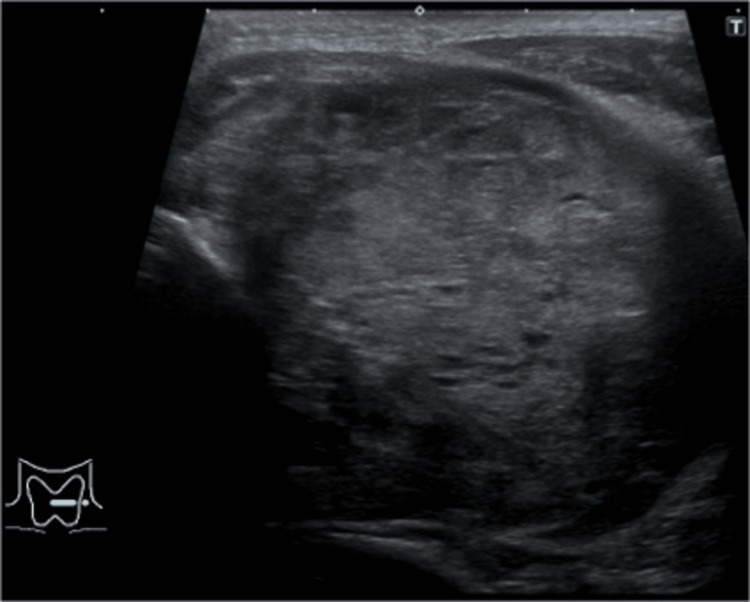
Cervical ultrasonography of the left thyroid lobe. In the left lobe of the thyroid, a solid nodule was observed with irregular shape, indistinct margins, and slightly heterogeneous internal hypoechogenicity.

On initial examination, a hard mass was palpated in the left anterior neck. Cervical ultrasonography revealed a tumor occupying the left lobe of the thyroid gland (Figure 1). Laryngeal fiberscopy showed no vocal cord paralysis. Laboratory findings were significant for hypothyroidism and an elevated thyroglobulin (Tg) level of 24,400 ng/mL (Table 1).

**Table 1:** Laboratory data of thyroid function tests and tumor markers. FT₃: free triiodothyronine, FT₄: free thyroxine, TSH: thyroid‑stimulating hormone, Tg: thyroglobulin, TgA: thyroglobulin antibody.

Investigation	Result	Reference value
FT3	1.77 pg/mL	2.3-4.0 pg/mL
FT4	0.32 ng/mL	0.9-1.7 ng/dL
TSH	161.2 μIU/mL	0.5-5.0 μIU/mL
Tg	24,400 ng/mL	<35.1 ng/mL
TgA	35.4 IU/mL	<19.3 IU/mL

**Figure 2:**
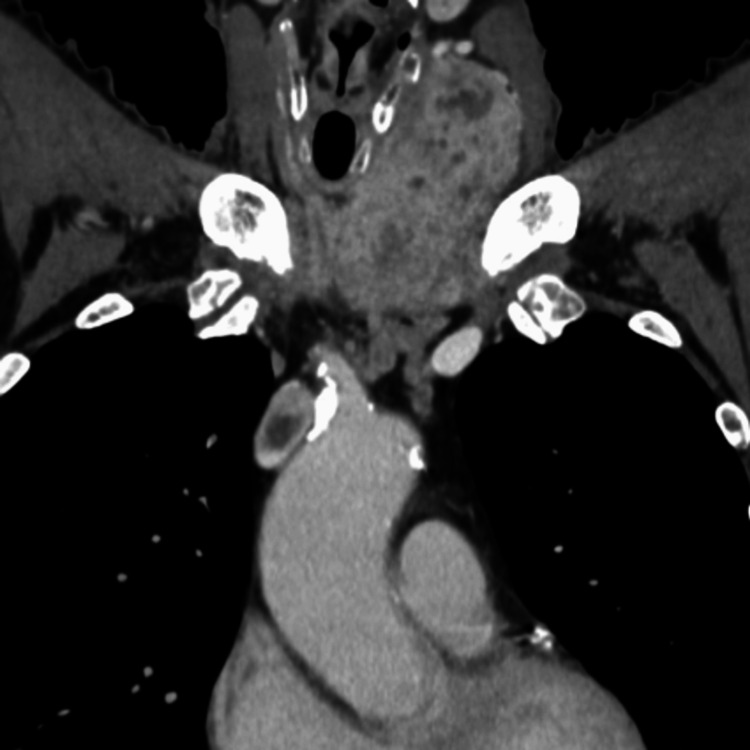
Contrast-enhanced CT showing a tumor measuring 84 × 62 × 54 mm that extends from the left thyroid lobe into the isthmus.

**Figure 3:**
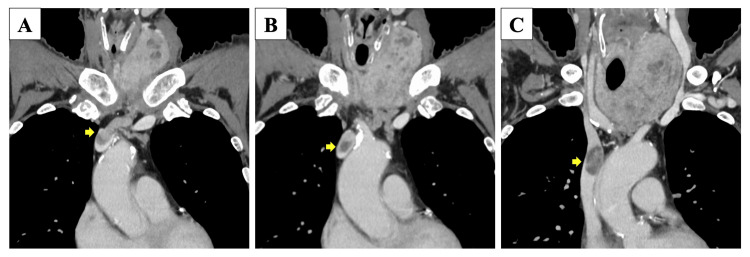
(A-C) Contrast-enhanced CT showing the thyroid tumor extending through the inferior thyroid vein, left brachiocephalic vein, and into the superior vena cava (SVC), suggesting tumor extension into the SVC (arrow).

**Figure 4:**
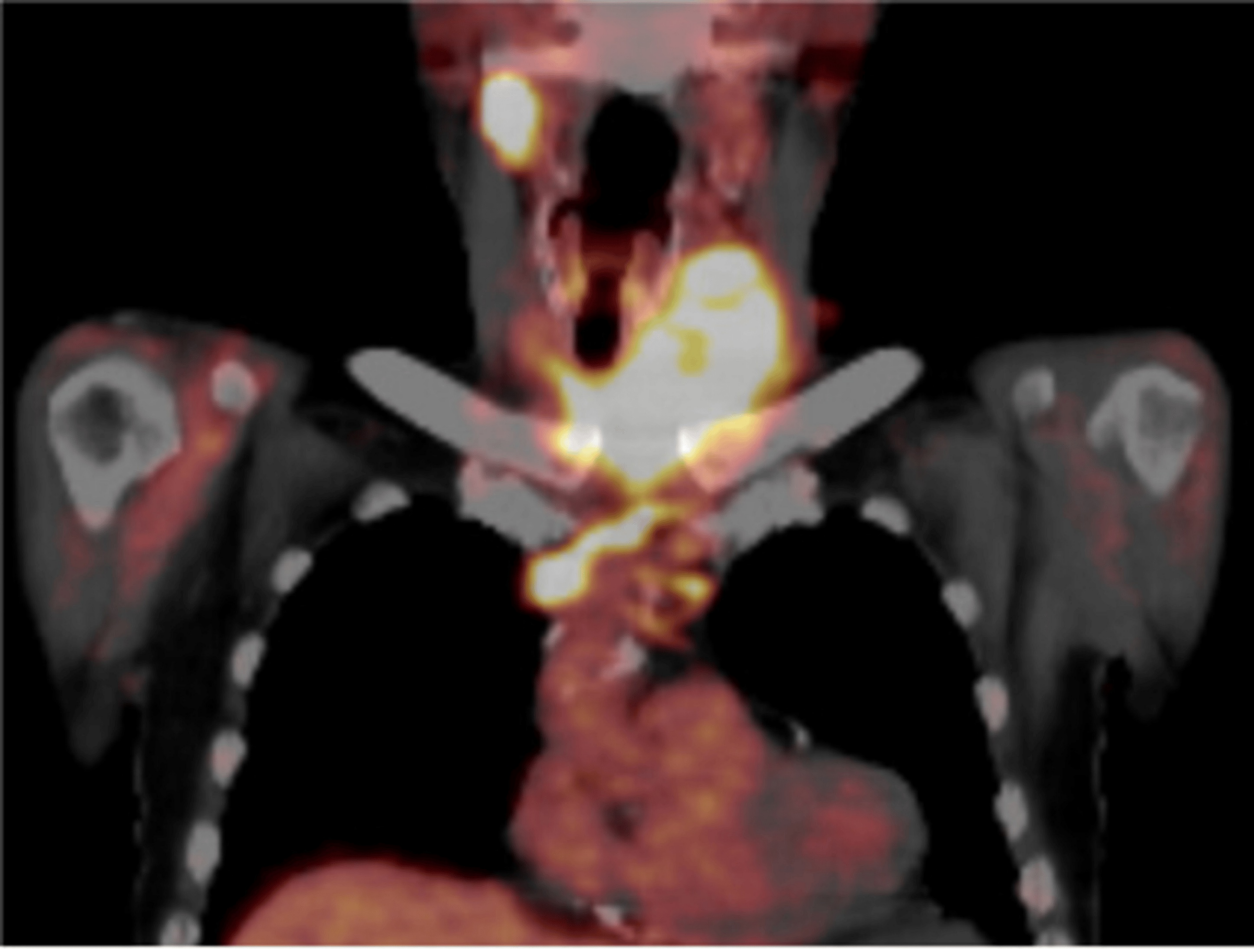
PET-CT of the thyroid tumor revealing an abnormal FDG uptake in the left thyroid lobe and in the tumor extending along the vessels, confirming intravascular invasion.

**Figure 5:**
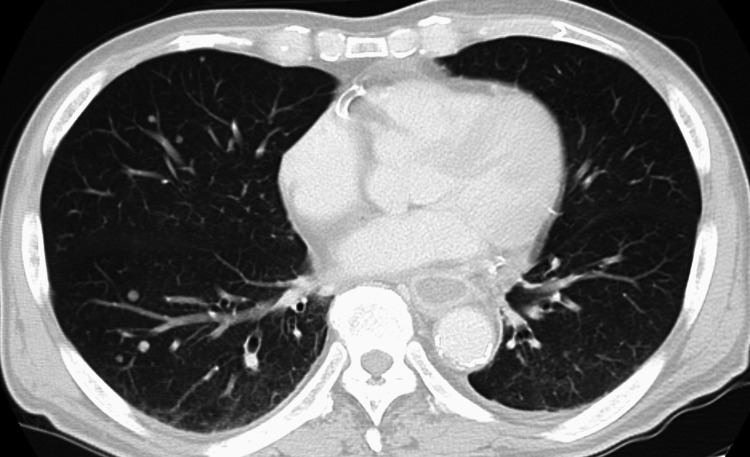
Plain chest CT detecting multiple pulmonary metastases.

Contrast-enhanced CT showed a tumor extending from the left thyroid lobe into the isthmus (Figure 2), with tracheal compression but no signs of tracheal invasion. The tumor measured 84 × 62 × 54 mm on CT. Multiple enlarged lymph nodes were observed in the bilateral neck and mediastinum. A continuous, heterogeneous, poorly enhanced region was identified, extending from the thyroid tumor through the inferior thyroid vein, left brachiocephalic vein, and into the SVC, suggesting tumor extension into the SVC (Figure 3). However, invasion into the SVC wall was considered unlikely. Furthermore, the venous tumor thrombus showed uptake on Positron emission tomography-computed tomography (PET-CT) (Figure 4). Multiple nodules of various sizes, suspicious for pulmonary metastases, were noted in both lungs (Figure 5). The patient did not present with SVC syndrome.

**Figure 6:**
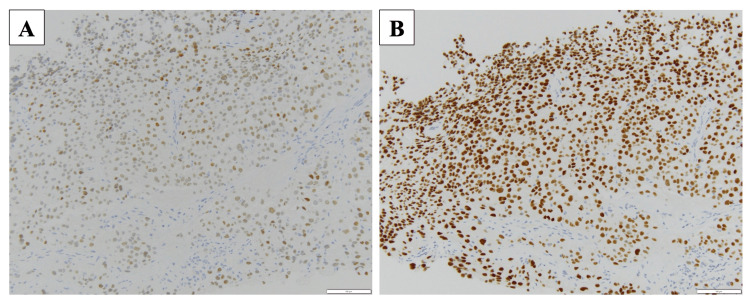
Immunohistochemistry (IHC) of the thyroid tumor. IHC was positive for (A) TTF-1 and (B) PAX-8.

A needle biopsy of the thyroid tumor led to a suspected diagnosis of PDTC. Immunohistochemistry was positive for TTF-1 and PAX-8 (Figure 6), negative for Napsin A and thyroglobulin, and showed a wild-type p53 pattern. The Ki-67 labeling index was 3%. The diagnosis was PDTC (cT4bN1bM1, Stage IVB), with cervical lymph node metastasis, pulmonary metastasis, and tumor extension into the SVC.

Although early surgical treatment initiation was considered, endoscopic therapy was required for stenosis of a duodenal ulcer scar. Consequently, a joint operation with our department and the Department of Cardiovascular Surgery was performed four months after the initial visit. The planned procedure was a total thyroidectomy, bilateral neck dissection, and removal of the intravascular tumor thrombus. Vascular replacement was also planned in case resection of the SVC or brachiocephalic vein was necessary due to tumor invasion. Additionally, the plan was to perform a tracheostomy if intraoperative findings suggested a high risk of bilateral vocal cord paralysis.

Surgical findings

Under general anesthesia, the patient was equipped with an intraoperative nerve monitoring (IONM) system, transesophageal echocardiography, and was prepared for direct current defibrillation (DCD). A U-shaped skin incision was made, and a conservative neck dissection of levels II-IV was performed on the right side. Subsequently, a conservative neck dissection of levels II-IV was performed on the left side.

The procedure then moved to a total thyroidectomy. The right thyroid lobe showed no adhesion or enlargement, and it was easily dissected after identifying and preserving the recurrent laryngeal nerve. The left thyroid lobe was diffusely enlarged but showed no adhesion to the trachea or surrounding muscles. A tumor thrombus was found extending from the thyroid into the left middle thyroid vein. As shown in Figure 3, this was transected at its junction with the left internal jugular vein, and the vessel was sutured. The left recurrent laryngeal nerve could not be identified intraoperatively.

**Figure 7:**
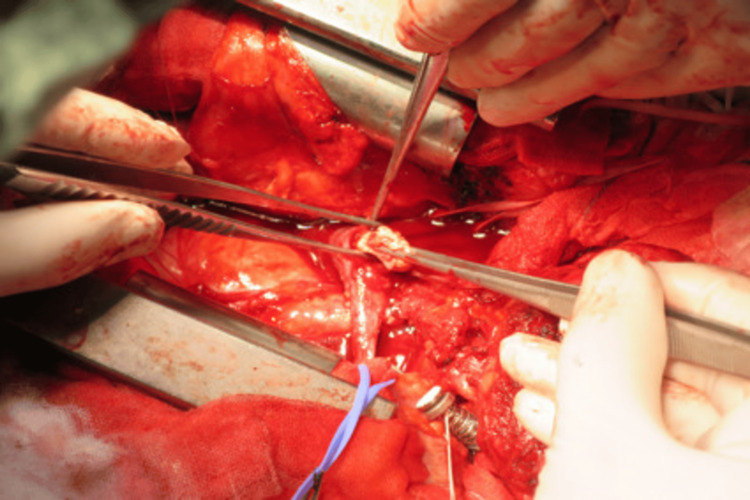
Intraoperative photograph. After incising the left brachiocephalic vein, the intravascular tumor was removed.

**Figure 8:**
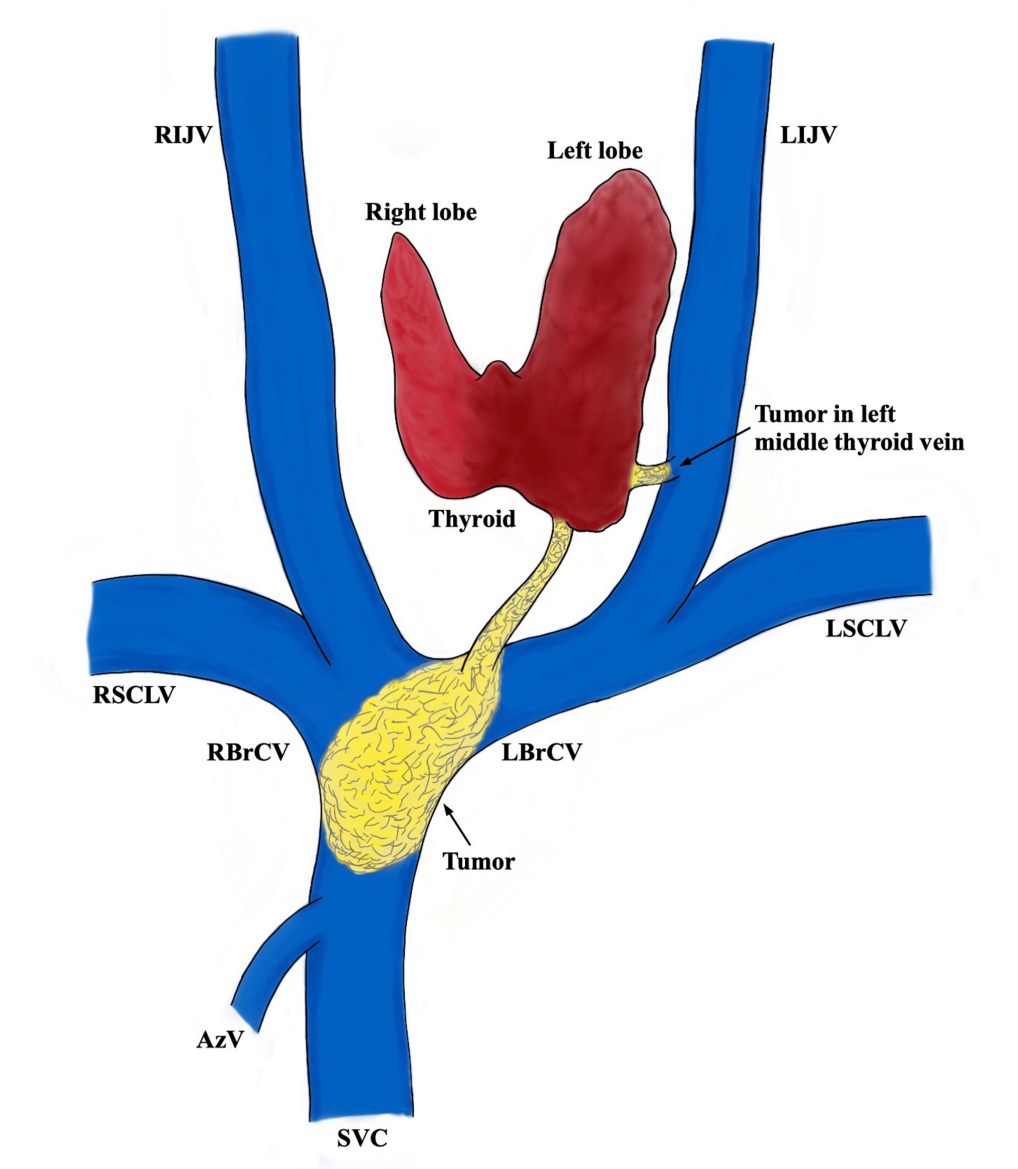
Schematic illustration of tumor progression from the thyroid into the central venous system. The tumor extended through the middle and inferior thyroid veins into the right and left brachiocephalic veins and subsequently reached the superior vena cava (SVC). This illustration was created by the authors using original operative findings. RIJV: right internal jugular Vein, LIJV: left internal jugular vein, RSCLV: right subclavian vein, LSCLV: left subclavian vein, RBrCV: right brachiocephalic vein, LBrCV: left brachiocephalic vein, AzV: azygos vein, SCV: superior vena cava.

The cardiovascular surgery team then proceeded with the thoracotomy through the 4th-5th intercostal space. After a pericardiotomy, both brachiocephalic veins were exposed. A tumor was confirmed to be extending from the lower pole of the left thyroid lobe, through the left inferior thyroid vein, and into the SVC. To prevent pulmonary embolism from the tumor, the SVC was clamped, and a venotomy was made in the left brachiocephalic vein. This allowed for clear visualization of the internal tumor thrombus (Figure 7), which was then resected up to the SVC. During removal, there were no findings suggestive of invasion into the vessel wall. Finally, the thyroid gland and paratracheal fatty tissue were removed en bloc (Figure 8). Before closure, the response of the right recurrent laryngeal nerve was confirmed with IONM; therefore, a tracheostomy was not performed.

Histopathological findings

**Figure 9:**
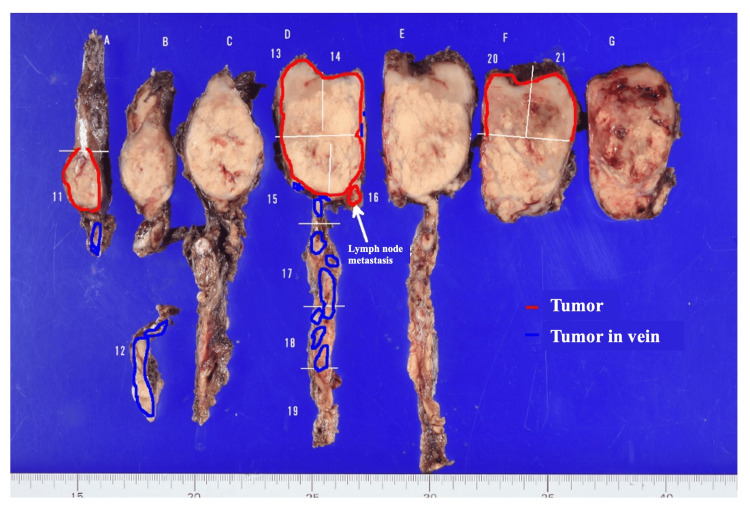
Macroscopic photo of a pathology specimen. The entire thyroid gland was replaced by the tumor, with extracapsular extension present, but no invasion into the anterior neck muscles was observed.

**Figure 10:**
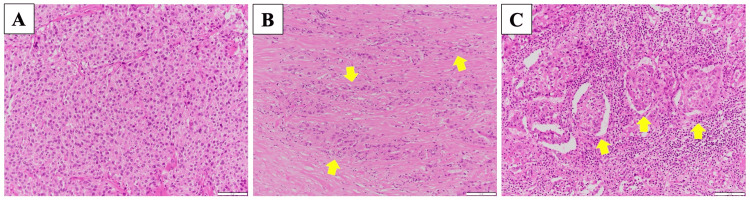
Pathological findings of the surgically resected specimen. A (A) solid, (B) trabecular, and (C) insular (arrow) growth pattern, which is characteristic of poorly differentiated thyroid carcinoma in the Turin classification, was observed.

The entire thyroid gland was replaced by the tumor (Figure 9), with extracapsular extension present, but no invasion into the anterior neck muscles was observed. Additionally, no extranodal extension was found in the metastatic lymph nodes. According to the Turin criteria, a solid/trabecular/insular (STI) growth pattern was observed (Figure 10). There was an absence of the conventional nuclear features of papillary carcinoma, and convoluted nuclei were present. Based on these findings, a diagnosis of PDTC was made.

Postoperative course

Although we had determined with IONM that there was no right vocal cord paralysis, we opted for delayed extubation and continued mechanical ventilation to mitigate the risk of airway stenosis due to laryngeal edema, which could result from the intraoperative SVC clamping and prolonged surgery near the larynx. The patient was successfully extubated on postoperative day 5. Left vocal cord paralysis was observed, but right vocal cord paralysis was not. No laryngeal edema was present. The patient was discharged on postoperative day 9.

To assess eligibility for RAI therapy, a 131I scintigraphy was performed. However, no lesion uptake was observed, and the tumor was deemed RAI-refractory. Treatment with lenvatinib was initiated three months postoperatively. The patient later developed Grade 3 hand-foot syndrome, and lenvatinib has been continued with intermittent dose interruptions. At the 18-month postoperative CT follow-up, there are no signs of local recurrence in the neck, and the multiple pulmonary metastases remain stable (stable disease). Thyroglobulin levels have remained low without any increase.

## Discussion

PDTC is a rare thyroid cancer of follicular cell origin, first recognized as a distinct entity by the WHO in 2004 [1,2]. The Turin criteria, which established the current histological standards for diagnosing PDTC, were published in 2007 [1,3]. Three histological criteria for diagnosis were proposed in 2007 and subsequently adopted in the fourth edition of the WHO classification, published in 2017. These criteria are (1) the presence of STI growth pattern, (2) the absence of the typical nuclear features of papillary thyroid carcinoma, and (3) at least one of the following three features: convoluted nuclei, ≥3 mitoses per 10 high-power fields, or tumor necrosis.

The incidence of PDTC varies by geographical region, accounting for less than 1% of all thyroid cancers in Japan, 2%-3% in North America, and up to 15% of diagnosed cases in Northern Italy [7]. This variation in incidence is thought to be due to differences in environmental factors and histopathological interpretation. In PDTC, regional lymph node or distant metastases are common at diagnosis, with approximately 70% of patients presenting with locally advanced disease, and the median five-year survival rate is 50%-60% [8]. Distant metastases eventually develop in 85% of cases during follow-up and are responsible for the majority of disease-related deaths [1]. Poor prognostic factors include male sex, older age, extrathyroidal extension, a high mitotic rate, and the presence of distant metastases [9,10]. Our case is considered to belong to a poor-prognosis group due to the presence of male sex, older age, extrathyroidal extension, and distant metastases.

The formation of a tumor thrombus in a major vein is rare in thyroid carcinoma, with a reported incidence of 0.2%-3.8% [11-13]. Intravenous tumor thrombi in thyroid carcinoma are known to arise from direct invasion of the primary tumor into the thyroid venous system, the adjacent internal jugular vein, or from metastatic lymph nodes. Only a few reports have described patients with thyroid carcinoma where the tumor thrombus extended beyond the internal jugular vein into the brachiocephalic vein, SVC, or intracranial sigmoid sinus [4-6]. While venous tumor thrombi in thyroid cancer are often asymptomatic, patients with a thrombus in the SVC frequently develop SVC syndrome [6].

The standard treatment for PDTC is surgical resection, with the goal of complete tumor removal, followed by postoperative administration of RAI or a multi-kinase inhibitor (MKI) [14,15]. In our case, we performed a total thyroidectomy and removal of the intravascular tumor for the primary lesion and considered postoperative RAI for the distant metastases. However, the tumor extended directly from the thyroid or via the thyroid veins into the SVC. Therefore, the feasibility of surgical resection was carefully considered, given the significant invasiveness and risk of complications associated with removing the intravascular tumor. Thomas et al. [15] and Zerhouni et al. [16] have reported the utility of contrast-enhanced CT in diagnosing major vein thrombosis. Typical imaging findings of a tumor thrombus include vessel expansion, an intraluminal mass that enhances similarly to the primary tumor, good enhancement of the vessel wall, and swelling of adjacent soft tissues. In our case, the preoperative contrast-enhanced CT showed that the tumor extending into the inferior thyroid vein had an attenuation similar to the primary thyroid tumor. However, within the brachiocephalic vein and SVC, contrast enhancement was observed between the periphery of the tumor and the vessel wall. Furthermore, no clear evidence of vessel expansion was seen proximal to the tumor extension in the SVC. Based on these imaging findings, we concluded that the tumor extending into the brachiocephalic vein and SVC was unlikely to have invaded the vessel wall and that it could be resected while preserving these veins. We planned for combined resection and replacement with a vascular graft if intraoperative findings revealed tumor invasion into the brachiocephalic or SVC. Consistent with the preoperative CT predictions, intraoperative findings showed no evidence of tumor invasion into the walls of the brachiocephalic vein or SVC. This allowed for the complete removal of the intravascular tumor simply by performing a venotomy on the brachiocephalic vein.

As mentioned by Kawano et al. [17], guidelines for the management of venous tumor thrombi in thyroid carcinoma have not yet been established. For tumor thrombi in the internal jugular vein, thrombectomy or segmental resection of the vein is often performed [18,19]. When the tumor thrombus extends into the brachiocephalic vein or SVC, a sternotomy is required in most cases to obtain an adequate surgical field. It is also necessary to consider whether to perform a thrombectomy or to resect the affected venous segment with subsequent venous reconstruction. Since many thyroid carcinomas are histologically well-differentiated tumors, there are reports that a tumor thrombus may not invade the vessel wall, allowing for thrombectomy without venous resection [20].

Koike et al. [4] pointed out differences in intravascular extension based on histopathological type, stating that papillary carcinoma tends to invade adjacent vessels from metastatic lymph nodes, whereas follicular carcinoma directly invades and extends within the vessels. PDTC often has a follicular structural component, and since the frequency of RAS mutations (18%-27%) is higher than that of BRAF mutations (0%-13%), it is thought that many PDTCs originate from follicular tumors [14]. In our case, however, neither RAS nor BRAF mutations were detected. Considering the patterns of intravascular extension described by Koike et al. [4], the surgical findings in our case suggest the possibility that the PDTC originated from a follicular carcinoma.

If venous invasion prevents thrombectomy, resection of the brachiocephalic vein or SVC and subsequent vascular replacement become necessary, and preparations for this should be made. In this case, replacement with a vascular graft was planned in case combined resection of the major vessel was necessary. When the tumor thrombus is confined to the brachiocephalic vein, the use of an SVC filter may be useful for preventing pulmonary embolism. However, if the tumor extends into the SVC, filter placement becomes difficult [17], and options such as SVC clamping or cardiopulmonary bypass are considered. We considered performing a tracheostomy in this case but decided against it, concluding that if bilateral vocal cord paralysis could be avoided, forgoing tracheostomy would reduce the risk of surgical site infection, especially since there was no lesion in the right thyroid lobe or the area of right paratracheal dissection.

## Conclusions

This is a rare report of the successful surgical resection of PDTC, a rare subtype of thyroid cancer, with extension into the superior vena cava. Accurate preoperative CT interpretation and close collaboration with cardiovascular surgeons are crucial for surgical planning.
